# An empiric tool to identify Kenyans living with HIV who will have unsuppressed viremia 18 months following treatment initiation to guide differentiated care models

**DOI:** 10.1371/journal.pone.0271520

**Published:** 2022-07-19

**Authors:** Njambi Njuguna, Nelly Mugo, Omu Anzala, Marianne Mureithi, Elizabeth Irungu, Joyce Wamicwe, Jared M. Baeten, Renee Heffron

**Affiliations:** 1 Department of Medical Microbiology, University of Nairobi, Nairobi, Kenya; 2 FHI 360, Nairobi, Kenya; 3 Kenya Medical Research Institute, Nairobi, Kenya; 4 Partners in Health and Research Development, Thika, Kenya; 5 Departments of Epidemiology and Global Health, University of Washington, Seattle, Washington, United States of America; 6 Ministry of Health, Nairobi, Kenya; 7 Gilead Sciences, Foster City, CA, United States of America; Emory University, UNITED STATES

## Abstract

**Background:**

With the global push towards universal access to Antiretroviral Treatment (ART), patient numbers are increasing, further straining already under-resourced healthcare systems in sub-Saharan Africa. A simple scoring tool could be useful in optimizing differentiated service delivery by identifying individuals likely to have unsuppressed viral load.

**Methods:**

Using existing data of patients accessing ART at public health facilities that were extracted from the Kenya Electronic Medical Record (KenyaEMR) and standard methods of developing a clinical prediction tool; we created and validated a risk scoring tool to identify persons likely to be virally unsuppressed at 18 months post-ART initiation. Data from the KenyaEMR were cleaned, merged and reviewed for completeness. We utilized multivariate modelling to determine key predictors of viral load suppression that could be measured in clinical settings.

**Results:**

We assessed clinical reports of 3,968 patients on ART who had been on ART for at least 18 months and had at least one viral load result and were ≥ 18 years old. Of these, the majority (81%) were virally suppressed 18 months post-ART initiation. The final risk score included age, sex, body mass index at HIV diagnosis, number of years of formal education, disclosure status, and duration of time between HIV diagnosis and initiating ART. The maximum risk score was 78; a risk score of ≥22 was associated with unsuppressed viral load (>1000copies/mL). The area under the curve (AUC) for the probability of the risk score to correctly predict unsuppressed viral load was 0.55 (95% CI: 0.52 to 0.56). Internal and external validation showed similar predictive ability.

**Conclusions:**

Routinely collected variables in a public HIV clinic medical record predicts, with modest accuracy, individuals likely to have unsuppressed HIV viremia 18 months after they initiate ART. The use and application of this tool could improve and complement efficiency in differentiated care models for patients on ART.

## Introduction

Approximately 1.5 million people in Kenya were living with HIV at the end of 2020 and the majority were using antiretroviral therapy (ART). Sustained adherence to ART with subsequent viral suppression has community HIV prevention benefits, health benefits for the person living with HIV, and HIV prevention benefits for HIV-negative sexual partners [1,2]. In Kenya, the prevalence of viral suppression among adults is estimated at 71.6% with wide variation from 39.7%-84.0% across counties [3]. Delivery of ART and support to people living with HIV (PLHIV) in sub-Saharan Africa (SSA), including Kenya, is hindered by several challenges, including scarcity of skilled healthcare workers, congestion in health facilities, and lack of resources to deliver and monitor treatment options to affected persons [4–6]. Globally and locally, there has been a concerted push to implement the World Health Organization’s ‘test and start’ approach where ART is provided to all HIV infected persons immediately after diagnosis resulting in an increased number of persons seeking HIV services. Adoption of this approach, however, has not been matched with an expansion of facilities and resources that would support the necessary level of delivery and monitoring of ART. This scenario sets up a situation of sustained pressure for expanded ART use with inadequate infrastructure to support individuals taking these drugs, resulting in increased numbers of individuals with poor adherence and subsequent gaps in medication performance.

In Kenya, PLHIV are typically offered ART and are taken through ART counselling sessions at the first visit after HIV diagnosis. They are then reviewed 2 weeks following ART initiation and then every 1–3 months thereafter based on their adherence patterns. Differentiated care models (DCM) have been introduced to provide scheduling options, with increased time interval between visits from 3 to 6 months with multi-month ART dispensing either at the healthcare facility or community sites. These models have been invaluable in reducing the burden on HIV treatment centers while maximizing the use of available human resource capacities [7]. Less frequent clinic visits have been observed to improve adherence to ART, promote patient autonomy and relieve the burden on patients and healthcare providers [8,9]. In Kenya, DCM guidelines became available in 2016 but DCM schedules were only recommended for stable patients who have been on ART for at least one year and have sustained viral suppression (<1000 copies/ml) [10]. This latest shift in implementation demonstrates the wide acceptance of DCM but also highlights a need for tools that can help providers to identify individuals that may need extra support with ART use so that they can have more frequent touch-points with provider and clinic staff. We used routinely collected empirical data from the Kenya Electronic Medical Record (KenyaEMR) to develop a tool that predicts people likely to have unsuppressed HIV viral load 18 months after ART initiation who would most benefit from maintaining routine, rather than less frequent, ART schedules.

## Methods

We used individual-level data from PLHIV accessing care from HIV treatment centers within Kenya whose clinical records were available in the KenyaEMR system. We created a prediction model and scorecard to identify people most likely to be virally unsuppressed 18 months after ART initiation, for whom more frequent clinic visits may be recommended.

### Study population and procedures

The KenyaEMR system is implemented in more than 300 HIV treatment centers within Kenya by the National AIDS and STI Control Program (NASCOP). Typically, some facilities key in data directly into the KenyaEMR while some collect records on paper forms and abstract them into the system. Records are then uploaded weekly onto the NASCOP data warehouse where they are stored. The purpose of the system is to create digital clinical records for patients accessing HIV care through these facilities. Through standardized electronic forms, the KenyaEMR collects a comprehensive set of variables that are captured in the Ministry of Health comprehensive care visit card (MOH247 form) which is used in all HIV treatment centers countrywide.

For this analysis, medical record data captured between January 2015 and August 2017 were used. De-identified data were received from the National AIDS and STI control Program (NASCOP) KenyaEMR system as two datasets: a) socio-demographic data which were captured through client intake forms at the HIV treatment centers, and b) viral load data including dates when samples were drawn and corresponding results captured through the MOH247 form. The datasets were merged to create one master dataset and duplicates were removed. Records were excluded if they did not have at least one viral load measurement ≤18 months post-ART initiation. Data were collected up to 19 months post-initiation to account for programmatic delays at month 18 visit which are common in clinical practice. In addition, the analysis was restricted to people aged ≥18 years because younger populations may need a different set of monitoring criteria due to child/adolescent dosing schedules and other features of childhood and adolescence. Analysis was restricted to records after January 2015 when routine viral load testing was scaled up nationally and no longer limited to use in cases suspected of treatment failure.

External validation was conducted using data from the Partners Demonstration Project, an open label evaluation of PrEP effectiveness among 1013 HIV serodiscordant couples that ran from November 2012 to December 2015 [11]. Couples in the project were in stable mutually- disclosed relationships and received counseling and HIV prevention services at 4 research clinics in Kenya and Uganda. Partners living with HIV were followed quarterly with semi-annual viral load and CD4 measurements and comprehensive counselling about the benefits of initiating ART.

### Risk score variables and statistical analysis

Our goal was to design a risk scoring tool that delineates between people likely and unlikely to be virally suppressed 18 months after ART initiation using 3 to 10 predictors routinely captured during client intake appointments. The primary outcome was unsuppressed viral load, defined as ≥1000 copies/mL, at 18 months after ART initiation. For persons with multiple viral load results, we used the first viral load documented within the 18-month window. We used univariate logistic regression models to identify enrollment characteristics, medical and HIV treatment characteristics, and family characteristics associated with unsuppressed viral load after 18 months on ART. These included demographic characteristics (age, gender, education level, marital status), clinical factors (body mass index, baseline CD4 count), and behavioural factors (disclosure of HIV status, presence of treatment supporter). The list of variables considered were derived from data routinely collected during clinic visits of HIV patients, particularly focusing on data collected at enrollment into the ART clinic. Continuous variables were grouped into categories based on clinically relevant cutoffs. All factors associated with unsuppressed viral load in univariate analysis with a p-value of <0.10 were combined into a multivariate logistic regression model and a stepwise sequence selection procedure used to identify the combination of variables that best predicted unsuppressed viral load with the lowest Akaike Information Criterion (AIC). To derive the score value for each level of each predictor, each coefficient from the final multivariate logistic model was divided by the lowest coefficient among all predictors and rounded to the nearest integer. To improve the precision of the predictive score, we ran the model through multiple iterations using adjusted grouped categories for continuous variables and tested various cutoff points for each predictor. We used receiver operating curves to estimate the area under the curve (AUC) and to analyze the predictability of the final multivariate model and score and determine the score cutoff that best balances sensitivity and specificity.

### Validation

We used a 10-fold cross validation technique to internally validate our model. External validation was done by applying the risk scoring tool to baseline data from each participant living with HIV in the Partners Demonstration Project and calculating their score. We then looked across scores to determine if the score accurately predicted unsuppressed viral load among individuals in the Partners Demonstration Project. All analyses were conducted using STATA version 13.1 (College Station, TX, USA).

### Human subjects protection

The Kenyatta National Hospital Ethics Review Committee approved the study protocol (P66/7/09/2016). The Partners Demonstration Project protocol was approved by the Kenya Medical Research Institute ethical review board and the University of Washington Human Subjects Review Committee.

## Results

### Population

**Fig 1:**
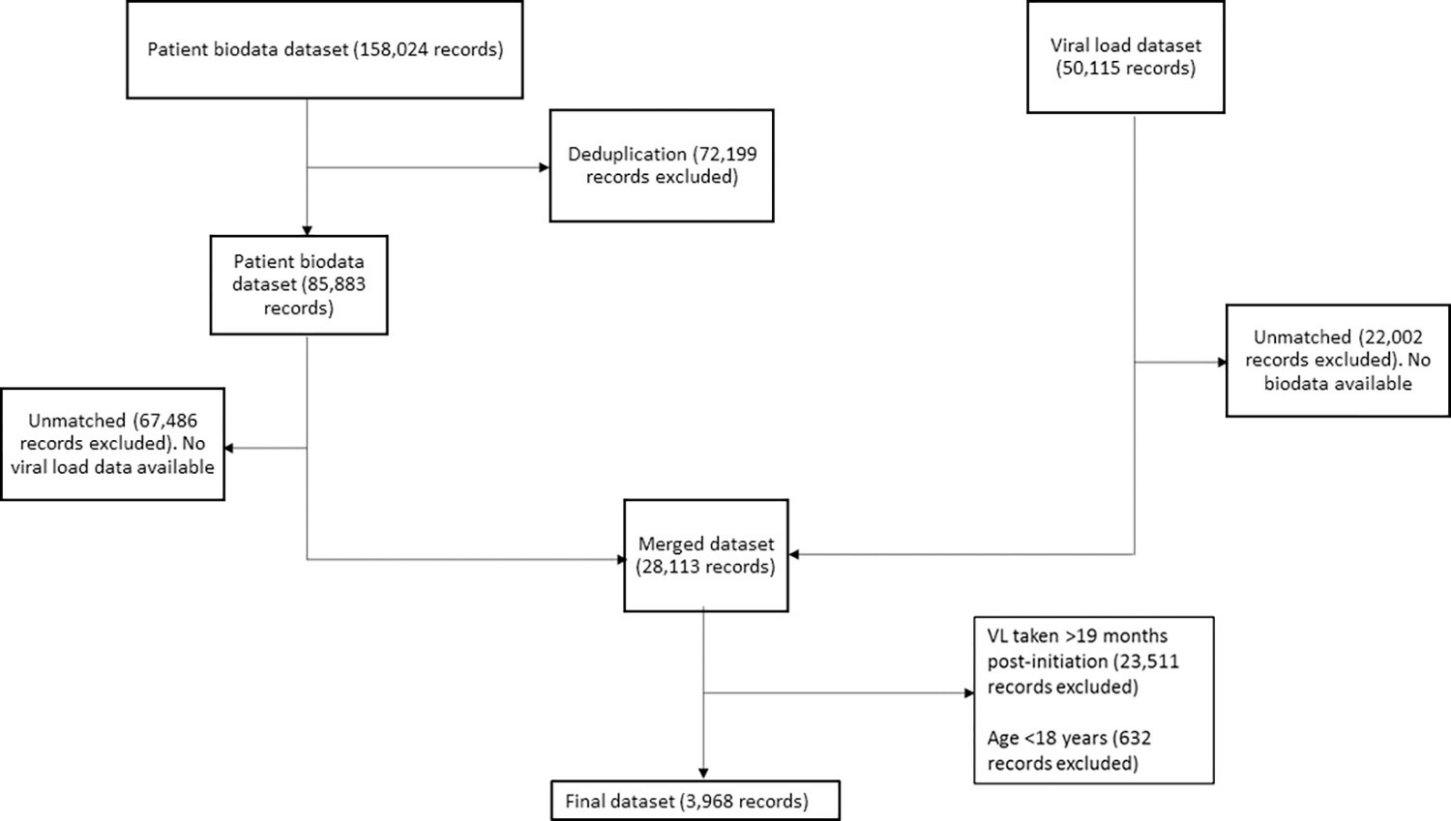
Flow diagram of data deduplication and cleaning to derive final dataset for scoring tool.

The datasets were cleaned to remove duplicates and merged to exclude records of persons who did not have viral load data or biodata resulting in a total of 28,113 people retained. Of these, records were excluded if the person was aged <18 years (632 people) or had their first viral load measurement conducted more than 18 months post-ART initiation (23,511 people). The final dataset for score derivation included data from 3,968 individuals (Fig 1). Of these, 73.2% of all the individuals were female, the median age was 36 (interquartile range [IQR]: 36–37), and 63% were married or cohabiting with a partner. A majority had ≤8 years of formal schooling and had not disclosed their HIV status to others (Table 1). In the Partners Demonstration Project, the majority of HIV positive participants were female, married with a median age of 28 years (interquartile range [IQR]: 27–28). Experience with education was similar to clients in the KenyaEMR.

**Table 1:** Participant characteristics.

	Individuals with VL <1000 copies at 18 months (n = 3227)	Individuals with VL>1000 at 18 months (n = 741)
Age overall (median, IQR)	37 (36–37)	35 (33–36)
Sex at ART initiation (n, %)		
Female	2379 (73.7)	527 (71.1)
Marital status (n, %)		
Single	393 (12.2)	85 (11.5)
Married or cohabiting	2038 (63.2)	472 (63.7)
Separated/divorced	258 (8.0)	69 (9.3)
Widowed	411 (12.7)	87 (11.7)
Other/missing	127 (3.9)	28 (3.8)
Education level (n, %)		
Less than primary (0–7 years)	2131 (66.0)	459 (61.9)
Completed primary (8 years)	806 (25.0)	203 (27.4)
Secondary (> 8 years)	290 (9.0)	79 (10.7)
Body mass index (BMI) (n, %)		
Underweight (< = 17.99)	386 (12.0)	104 (14.0)
Normal (18–24.99)	2487 (77.0)	548 (78.8)
Overweight/obese (> = 25)	354 (11.0)	53 (7.2)
No treatment support partner (n, %)	3202 (99.0)	733 (98.9)
Had disclosed HIV status (n, %)	1130 (35.0)	223 (30.1)
CD4 count cells/ml (n = 811) (median, IQR)^a^	185 (173–199)	200 (162–221)

^a^ Missing data includes 2959 records, representing 75% of the total.

### Risk score model

**Fig 2:**
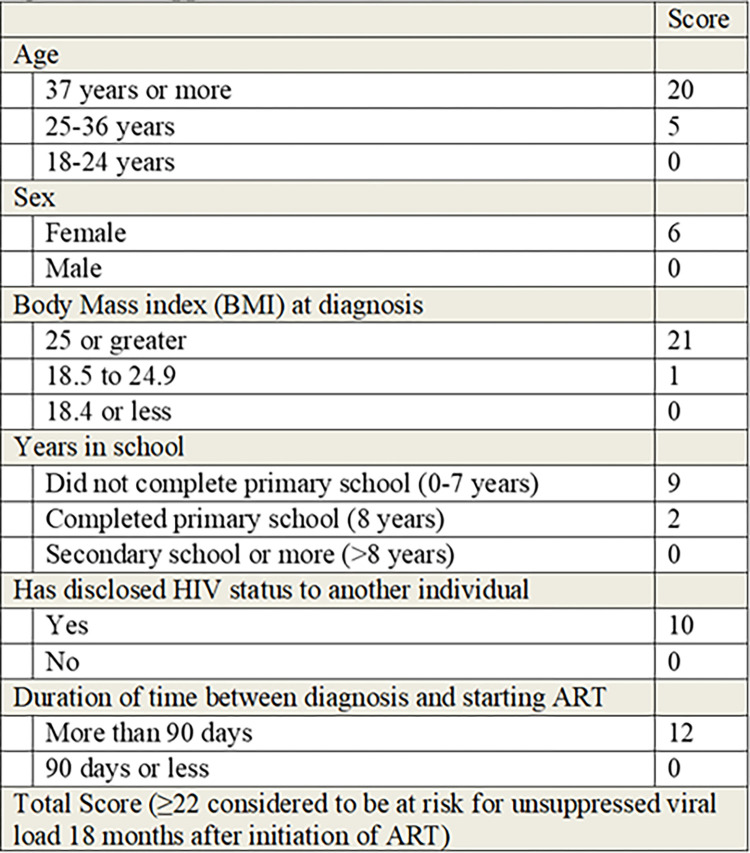
Unsuppressed viral load score.

Univariate analysis identified seven variables for inclusion in the multivariate analysis (Table 2). Of these, age, sex, education level, body mass index (BMI) at enrollment, disclosure of their status, and duration of time between HIV diagnosis and starting ART were retained in the final predictor model and contributed to the score card (Fig 2).

**Table 2:** Predictors of unsuppressed viral load at 18 months and calculation of risk score.

	Univariate analysis			Multivariate analysis		Stepwise multivariate analysis			
Variable	Odds Ratio	95% CI	p value	Odds Ratio	95% CI	Odds Ratio	95% CI	Regression coefficient	Risk score
**Age**									
18–24	ref		**0.0007**	Ref		Ref			
25–36	1.08	0.844–1.38		1.00	0.75–1.31	1.11	0.86–1.44	0.10	5
≥37	1.44	1.13–1.85		1.41	1.05–1.88	1.53	1.17–1.99	0.42	20
**Sex at ART initiation**									
Male	ref	0.95–1.36	**0.151**	Ref		Ref			
Female	1.14			1.12	0.91–1.38	1.13	0.94–1.37	0.13	6
**Marital status**									
single	ref		0.95						
cohabiting/married (monogamous/polygamous)	0.93	0.72–1.21							
separated/divorced/widowed	0.93	0.69–1.24							
other/missing	0.98	0.61–1.57							
**Education level**									
Secondary (>8 years)	ref		**0.096**	Ref		Ref			
Completed primary (8 years)	1.08	0.81–1.45		1.05	0.75–1.45	1.04	0.77–1.42	0.04	2
Less than primary (0–7 years)	1.26	0.97–1.65		1.21	0.89–1.64	1.2	0.90–1.58	0.18	9
**BMI**									
underweight (<18.5)	ref		**0.003**	Ref		Ref			
normal (≥18.5–24)	1.15	0.91–1.45		0.95	0.72–1.25	1.02	0.79–1.31	0.02	1
overweight/obese (≥25–29)	1.8	1.25–2.58		1.48	0.97–2.25	1.53	1.05–2.25	0.43	21
**Patient supporter**									
none	ref		0.43						
has treatment partner	0.72	0.32–1.59							
**Patient entry source**									
Voluntary counselling and testing center	ref		1						
other sources	1	0.84–1.18							
**Disclosure of status**									
no intervention	ref		**0.0102**	Ref		Ref			
disclosure/partner testing	1.25	1.05–1.49		1.22	1.01–1.49	1.23	1.02–1.47	0.20	10
**Days between diagnosis and starting ART (N = 3783)**									
0–90 days	ref		**0.0005**	Ref		Ref			
>90 days	1.34	1.14–1.58		1.33	0.88–2.01	1.29	1.09–1.52	0.26	12
**Duration of time from diagnosis to enrollment in HIV treatment centers (N = 3760)**									
0–7 days	ref		0.417						
8–14 days	1.37	0.90–2.08							
15–21 days	1.35	0.76–2.41							
22–60 days	1.08	0.76–1.52							
≥61 days	0.95	0.76–1.18							
missing	-	-							
**Days between enrollment into HIV treatment centers and starting ART (N = 3653)**									
0–14 days	ref		**0.0011**	Ref					
15–30 days	1.25			1.24	0.90–1.70				
31–90 days	0.92			0.93	0.71–1.21				
>90 days	1.4			1.02	0.66–1.57				

#### Model validation

We calculated the total risk score of each individual by summing the individual parameter scores determined in the final risk model. The AUC for the probability of the risk score to correctly predict unsuppressed viral load was 0.55 (95% CI: 0.52 to 0.56). Internal cross-validation showed the average AUC for 10 subsets analyzed was 0.55 (95% CI: 0.49–0.57), similar to the AUC of the full data set and indicating robust generalizability of the risk algorithm within the data set. A cutoff of 22 was determined to be optimal for balancing sensitivity and specificity when identifying persons with unsuppressed viral load. Using the cutoff risk score of ≥22, we identified 55% of people who were not virally suppressed from 63% of the population. For external validation, we applied our risk score to the Partners Demonstration Project. The AUC for the risk score applied to participants of the Partners Demonstration Project was 0.56 (95% CI: 0.48 to 0.57).

## Discussion

Results from this analysis have shown that baseline empirical data collected routinely in treatment centers within the Kenya public health system can be used with modest accuracy to predict unsuppressed viral load among individuals accessing ART 18 months after initiation. The predictors selected for our score are well-established variables that are routinely collected in clinical settings: age, sex, BMI, number of years of school, HIV disclosure status, and duration of time between HIV diagnosis and starting ART. These variables, in combination, were more predictive of unsuppressed viral load than each individual factor alone. Viral load suppression is mostly influenced by adherence to ART thus our risk score is likely to be useful in determining differentiated care models for persons who may be challenged with sustained ART adherence.

Similar risk scores have been established for patients accessing care in developed countries using a combination of self-reported adherence, clinical and bio-behavioural predictors [12,13]. However, in many instances, these tools are utilized prospectively to assess clients at their biannual or annual clinic visits and not at baseline. Our findings are relevant to programmatic implementation of differentiated care and support a more efficient model for ART delivery that tailors clinic schedules to the propensity for each patient to adhere to ART. Using this score to predict likelihood of unsuppressed viral load at the beginning of treatment and tailoring scheduled clinic visits to predicted viral load suppression will reduce the cost of treatment for people on care for the first 18 months. Currently, Kenya employs several evidence based interventions in supporting adherence including community based support groups and individual level support through peer champions or peer navigators [14]. Use of this empiric scoring tool will alleviate the burden on patients and reduce the strain on the public health system by allowing clinicians to reduce the time spent with patients predicted to have suppressed viral load while focusing intensified adherence support to those predicted to be virally unsuppressed. This is particularly relevant during the COVID-19 pandemic when face-to-face contact is recommended to be minimized when clinically possible.

Before the COVID-19 pandemic, patients with good ART adherence during the first year of ART use were seen with the same frequency as those experiencing adherence challenges and could only be switched to less frequent visits after one year of consistent ART use and confirmed viral load suppression, an approach that fails to maximize clinic resources and patients’ time. During COVID-19, however, DCM was more widely implemented in response to recommendations to reduce person-to-person contact. Patient appointment schedules were revised to include >3 months between clinic visits and to minimize contact with healthcare providers by distributing at least 3 months of ART for all patients regardless of their viral load or duration on treatment [15]. Thus, the patients and providers have fewer hesitations now with the reduced visit schedule. Use of an empirical scoring tool at ART initiation would help clinicians determine which patients were likely to be unsuppressed and focus resources on supporting those clients as needed.

We did not specifically investigate the causes of delay in viral load testing beyond 18 months for individuals whose data were excluded from this analysis as it was beyond the scope of this work. However, based on other published reports, delays are likely related to individual and systemic issues, with individual reasons including lack of treatment literacy leading to poor adherence to visit schedule, long distance to medical facility, lack of time off from work, and limited financial resources [16–18]. Systemic issues like prolonged waiting times at treatment centers, delayed turnaround times due to equipment breakdowns and difficulty in specimen transport have also been cited [18,19]. Different evidence-based mitigation measures to reduce these delays include community testing models where samples are collected at community level during peer support groups and transported to the facility for testing rather than waiting for the patients to come to the facility for testing [20]. In addition, adoption of point-of-care testing diagnostics by Ministry of Health and strengthening of laboratory referral systems would help in curbing instances of delays [21].

Though our study was derived from a large dataset of individuals from across Kenya, the AUC was 0.55, which is only a modest level of prediction. A limitation of our analysis was that behaviour, lifestyle and other factors known to influence adherence were not available to use since these are not collected on the MOH247 form. These factors may be important for the healthcare providers to consider in complement to the prediction adherence tool to select patients for differential visit scheduling. Understanding that adherence to ART is a nuanced behaviour, further work needs to include qualitative research to explore psychosocial factors influencing adherence and acceptability of our prediction tool. Another limitation of our study is that the dataset provided from the KenyaEMR had a high level of incompleteness in some variables resulting in high drop-off of total records available for use, which is similar to other studies using routinely collected national data, and an inherent source of bias. However, our validation models were based on a dataset with a very low level of missing data and thus was ideal in determining whether our scoring tool accurately identified individuals who fail to become virally suppressed within 18 months after initiating ART.

In summary, approaches that restructure ART delivery with a focus on the likelihood of adherence are needed to minimize clinic-based bottlenecks and improve patient care. Our risk score modestly predicts unsuppressed viral load and can be used to tailor patient visit schedules, counseling and adherence monitoring. Use of the model would yield a more efficient clinic system that identifies individuals early on who would benefit from more frequent interactions with healthcare providers, while supporting those with predicted suppression to maintain less frequent interactions and for the clinic to benefit from reduced clinic volume, decreased waiting time, and improved patient flow. However, further research would be needed to determine the feasibility and effectiveness of this score in clinical settings as well as the cost implications.
